# Leveraging the timing and frequency of patient decision aids in longitudinal shared decision‐making: A narrative review and applied model

**DOI:** 10.1111/hex.13531

**Published:** 2022-06-02

**Authors:** Lara R. LoBrutto, Gemmae Fix, Renda S. Wiener, Amy M. Linsky

**Affiliations:** ^1^ Center for Healthcare Organization and Implementation Research VA Boston & VA Bedford Healthcare Systems Boston Massachusetts USA; ^2^ Department of Medicine Boston University School of Medicine Boston Massachusetts USA

**Keywords:** decision aids, decision‐making, patient‐centred care, shared

## Abstract

**Introduction:**

Shared decision‐making (SDM) is intended to increase patient‐centredness of medical decision‐making for patients with acute and chronic conditions. Concurrently, patient decision aids (PtDAs) can supplement SDM by providing information to guide communication between patients and healthcare providers. Because of the prevalence of chronic conditions, where decisions may be extended or recurring, we sought to explore how effectively these tools have been leveraged in this context.

**Methods:**

We conducted a narrative review of the literature on both SDM and PtDAs, searching PubMed and Boston University's library database search tool for English‐language articles published from January 2005 until March 2021. Additional search terms focused on temporality. Drawing from our findings, we developed a combined framework to highlight areas for future research using the discussion of end‐of‐life decisions as an exemplar to illustrate its relevance to chronic care contexts.

**Results:**

After screening 57 articles, we identified 25 articles that fulfilled the inclusion criteria on SDM, PtDA use and temporality for chronic care. The literature on SDM highlighted time outside of the medical visit and opportunity to include outside decision partners as important elements of the process. PtDAs were commonly evaluated for process‐related and proximal outcomes, but less often for distal outcomes. Early evidence points to the value of comparative outcome evaluation based on the timing of PtDA distribution.

**Conclusion:**

Our review of the literature on SDM and PtDAs reveals less attention to the timing of PtDAs relative to that of SDM. We highlight the need for further study of timing in PtDA use to improve longitudinal SDM for chronic care. The model that we propose in our discussion provides a starting point for future research on PtDA efficacy.

**Patient or Public Contribution:**

Five patient consultants provided input and feedback on the development and utility of our model.

## 1 INTRODUCTION

Shared decision‐making (SDM) is a key component of patient‐centred care and is the recommended model for clinical decision‐making.^1^, ^2^ This model is characterized by patient and provider involvement in the process of evaluating options facing the patient, with a goal to incorporate patient preferences into healthcare choices.^3^, ^4^ Most implementation and evaluation of SDM have focused on activities of a single healthcare encounter.

Despite frequent application to acute care delivery or one‐time decisions, SDM is also highly relevant within the context of chronic care. Key features of SDM that may be particularly salient for chronic care include recognition of decision partners, time for patient reflection and occurrence of decision‐supporting activities external to the provider visit.^5^, ^6^, ^7^ However, a systematic review of SDM implementation found low levels of patient engagement in SDM for both chronic and acute conditions, indicating opportunity for improvement in both settings.^8^ Further, a thematic analysis noted that measurement challenges are rooted in the fact that SDM occurs over time.^9^

Patient decision aids (PtDAs) are valuable, prevalent tools to facilitate SDM^10^ and typically target a specific healthcare decision. They improve patient knowledge and patient‐centred outcome measures, with increased effect when designed at the appropriate health literacy level.^11^, ^12^ PtDAs can be distributed at various timepoints, including before, during or after a healthcare encounter; however, their use is often limited by provider‐level (e.g., disagreement with the content of materials) and system‐level barriers (e.g., lack of organizational support, limited provider time, poor continuity of care).^12^, ^13^, ^14^, ^15^, ^16^

Therefore, this paper aims to describe SDM and PtDAs in the context of care for chronic conditions. We begin by reviewing the literature on SDM and PtDAs, with particular focus on the temporal elements of each (i.e., the inclusion of concepts related to decisions occurring over time). Following this, we discuss our proposed intersection between the two concepts, illustrating how PtDAs might be timed for current models of SDM and later evaluated through this lens to determine optimal use. Finally, to demonstrate conceptual utility, we apply our framework to SDM about care towards the end of life, one of many contexts of SDM that unfolds over time.

## 2 METHODS

Following guidance promulgated by the Scale for the Quality Assessment of Narrative Review Articles,^17^ we conducted a narrative literature review. We searched in PubMed and Boston University's PRIMO (library database search tool) using a combination of the following key words and medical subject headings: ‘shared decision making’, ‘decision aid’, ‘decision tool’ and ‘educational materials’ to characterize the two concepts. To identify the literature on the timing of SDM and decision aid distribution, we combined the aforementioned search terms with the following: ‘systematic review’, ‘temporal’, ‘longitudinal’, ‘timing’, ‘continuum’, ‘sequence’, ‘pre‐visit’, ‘point‐of‐care’, ‘post‐visit’ or ‘after‐visit summary’. We reviewed all abstracts, including English‐language articles published from January 2005 until March 2021, that provided information about the current state of SDM and PtDAs and those that included discussion of timing as a variable for full‐text manuscript review. We also scanned reference lists of selected articles for additional relevant manuscripts. To find exemplar‐specific information, we used our original search terms combined with ‘palliative care’, ‘end‐of‐life care’, ‘life‐sustaining treatment’ and ‘code status’. For this search, we included only manuscripts published after January 2010 to reflect current practices. To enhance our understanding of how patients perceive timing of PtDA use, we consulted with five members of an expert advisory panel.^18^ Using videoconferencing technology, we conducted two listening sessions (with three and two participants, respectively). We asked panel members to consider real or hypothetical experiences using decision aids and provide opinions on the model and its utility. Notes were taken during the session, and focus groups were recorded for reference.

## 3 RESULTS: SDM

In contrast to acute care, decisions for chronic conditions are typically less time‐sensitive, involve subsequent opportunities to readdress and engage patients' social networks in decision‐making.^6^ Because ongoing conditions are often characterized by a long‐term relationship with a physician or other healthcare provider (HCP), development of self‐efficacy on the part of the patient and decisions being implemented beyond the clinical environment, the timeline of the decision‐making process inherently extends beyond one isolated event.^5^, ^7^ The original SDM model was developed in the context of acute care delivery; thus, Montori, Gafni and Charles^6^ argued that it must be adapted for successful provision of chronic care. Others have found fault with the narrow view of SDM as occurring solely within the medical visit and between the two parties of patient and provider.^19^, ^20^, ^21^, ^22^, ^23^, ^24^, ^25^ There is growing recognition that patients may consult outside individuals and materials before, between and/or after healthcare visits to encompass the multidimensional, lived reality of decision‐making.^5^, ^7^, ^26^, ^27^, ^28^

### 3.1 Beyond the patient‐provider dyad

Many SDM models emphasize the role of ‘decision partners’, such as spouses, family, friends, other HCPs and acquaintances, in a patient's medical decisions.^5^, ^7^, ^19^, ^21^, ^22^ Having a lay (i.e., nonmedical) person involved in decision‐making can be an asset.^19^, ^23^, ^24^ In some cases, partners may be present at a healthcare visit; however, more commonly, the patient may consult with these individuals outside of visits for advice, opinions or support before reaching a final decision with their HCP.^7^ Beyond this, it is simplistic to assume that patients enter a clinic as completely self‐contained beings; rather, their autonomy is ‘relational’, existing as members of complex social and interpersonal networks.^7^, ^19^, ^21^ Internet resources are also likely to inform SDM because of their ubiquity and ease of access,^7^, ^19^, ^25^ and they may introduce supporting or countering perspectives into the patient–provider encounter.^20^ These various inputs add complexity to what the patient brings to a medical decision, making it important for the HCP to assess the influence of these external factors on each patient's values.

### 3.2 Beyond the isolated clinical encounter

Closely tied to the role of outside influences, new SDM frameworks focus on the role of time before, between and after healthcare visits in the patient's trajectory of decision‐making. Notably, two models clearly emphasize this element of SDM. Clayman et al.^7^ conceptualize the healthcare visit as only one part of a four‐part SDM framework. In the ‘preparation stage’, the patient may conduct online searches and consult with trusted individuals. Following the ‘visit’ (second stage), ‘encounter processing’ (third stage) takes place, in which patients may continue to gather information and have conversations. The fourth and final stage, ‘feedback, continuation and resolution’, may be characterized by scheduling a follow‐up visit or continuing communication with the HCP before coming to a decision. The framework is represented cyclically, indicating that the process can restart once the patient returns for follow‐up.

In another model, Bomhof‐Roordink et al.^5^ envision the healthcare visit as occurring within the broader scope of patient and provider time, with elements of the decision‐making process occurring within and outside of the visit. Based on qualitative interviews with oncologists and patients, they reimagine the SDM process, which ‘extends to the world of the patient and is not confined to the space where oncologist and patient meet’.^5^ Both models illustrate that time outside of the visit is an integral component of SDM. Going forward, we refer to the explicit inclusion of temporality as *longitudinal shared decision‐making* (L‐SDM).

## 4 RESULTS: PtDAs

While the utility of decision aids for single‐timepoint‐based SDM is well supported,^10^ and the importance of L‐SDM for chronic care has been clearly demonstrated, the use of PtDAs to facilitate L‐SDM remains relatively unexplored. PtDAs are tools that may be used by HCPs to present options and guide patients to a decision. They vary widely in format and may be designed as brochures, videos or internet‐based information.^29^ They are often distributed before or during a clinical encounter. Previsit PtDAs typically provide comprehensive information about treatment options and are intended to be used before the visit so that the patient is primed for decision‐making.^12^, ^26^ In‐visit (also known as ‘point‐of‐care’, ‘encounter’ or ‘conversational’) PtDAs are often less complete because they are meant to promote patient–provider dialogue, sometimes through engaging visuals.^12^, ^14^, ^26^ Use of postvisit PtDAs, or take‐home materials, has also been documented,^27^, ^29^ but there is little information about their structure or effectiveness.

### 4.1 Benefits of using PtDAs

PtDAs promote SDM in many ways. Patients equipped with information are more likely to engage in their healthcare decisions,^4^ and PtDA use is correlated with decision‐making that aligns with patient values.^28^ According to a systematic review of provider satisfaction, clinicians found value in PtDAs as a reminder to engage patients, a facilitator of dialogue and a method of information‐sharing grounded in evidence that reduced providers' burden of educating patients.^30^ From a system‐level perspective, wide use of PtDAs can increase uniformity and promote adherence to standards of care.^31^

### 4.2 Outcome metrics to evaluate the effect of PtDAs

To date, there has been little comparative evaluation of PtDA outcomes based on variability in the timing of distribution (e.g., previsit vs. in‐visit). Instead, many systematic reviews and meta‐analyses have attempted to assess patient‐associated outcomes and, less often, provider‐associated outcomes. Commonly measured patient‐associated outcomes are both process‐related (e.g., occurrence of an SDM discussion, patient–provider communication) and proximal (e.g., patient knowledge and satisfaction, decisional conflict, decisional regret).^32^, ^33^, ^34^, ^35^, ^36^ There was less evidence for distal outcomes (e.g., health and behavioural health status, quality of life).^33^, ^34^, ^36^ Provider‐associated outcomes primarily assessed satisfaction, efficiency and personal and professional well‐being.^30^ One systematic review looked at healthcare system outcomes, including cost, cost‐effectiveness, consultation length and litigation rates.^36^ We identified two systematic reviews of PtDA for palliative care settings, which evaluated 12 and 16 tools, respectively. Both reviews assessed quality and demonstrated efficacy of the tools, and one review further evaluated specific process and proximal outcomes.^37^, ^38^

### 4.3 Timing of decision aid use

As a tool to facilitate SDM in general, PtDA placement in the decision‐making cycle can impact both patient and provider outcomes. Most studies evaluating PtDA effectiveness have not tested the optimal timing of distribution, and yet, a few studies did yield promising evidence. Although a systematic review comparing studies that used previsit versus in‐visit distribution found no differences in the mean patient knowledge or risk‐perception scores,^36^ another study compared pre‐visit and in‐visit distribution of the same PtDA and found significantly higher knowledge scores in the in‐visit group.^39^ Additionally, Hsu et al.^29^ noted variation in the timing, but not frequency, of PtDA use across six specialty areas, suggesting that optimal strategies are context‐dependent. The impact of the PtDA timing and frequency on providers' experience is also unclear. Whereas some studies have indicated that providers prefer previsit PtDAs due to time constraints and distribution barriers,^13^, ^27^, ^29^, ^40^ others suggest that in‐visit PtDAs are more effective at facilitating SDM.^12^, ^34^

## 5 RESULTS: PATIENT ADVISORY PANEL

Individuals noted value in receiving decision aids before, during or after a visit, suggesting variability in preferences despite potential benefits from all options. However, one noted that receiving information before a visit could be anxiety‐inducing if the proper supports were not provided. Another panel member said, ‘If this is going to be a [patient]‐centric solution, you gotta provide some flexibility so that the [patient] gets to choose the path they want to follow in gathering that information’. In other words, decisions about distribution points should incorporate patient preferences in addition to those of providers. It was suggested that opportunities for the patient and provider to correspond about the materials before or after their visit might enhance their value by allowing patients to ask questions of providers and giving providers the opportunity to prepare materials based on patient input. However, panel members also noted that patients may not read materials outside of a visit unless it was a very serious condition.

## 6 DISCUSSION: ENHANCING L‐SDM WITH OPTIMALLY TIMED DECISION AIDS

There is growing understanding of the extended timeline and involvement of decision partners in medical decision‐making for chronic conditions. Despite explicit inclusion of timing into SDM (i.e., L‐SDM), data on optimal timing of PtDA distribution remain relatively sparse. Early findings (see Section 4.3) suggest the importance of evaluating the timing of PtDA distribution in addition to content and single‐timepoint‐based outcomes.

**Figure 1:**
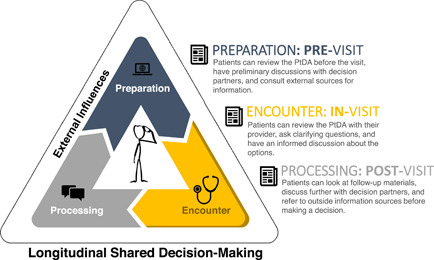
Mapping PtDA Timing onto L‐SDM. (1) Before the healthcare visit, there is opportunity for ‘preparation’, when a provider may share a ‘pre‐visit’ PtDA with the patient. (2) The healthcare visit or ‘encounter’ occurs next, during which the provider may use an ‘in‐visit’ PtDA to assist in an SDM conversation. (3) After the visit, ‘processing’ occurs, and can be aided by a ‘post‐visit’ PtDA. PtDA, patient decision aid; L‐SDM, longitudinal shared decision‐making.

We synthesized our narrative review findings to create a model intended to guide future research. In this model, we map three timepoints of potential PtDA distribution onto Clayman's first three phases of SDM (‘preparation’, ‘encounter’, and ‘processing’), excluding ‘feedback, continuation and resolution’, because it does not have a parallel PtDA distribution point (Figure 1). We view intentional timing and frequency of PtDA distribution as a mechanism by which to increase dialogue between the patient, HCP and outside influences. Advantages to the patient include ability to reflect upon and deepen understanding of their choices, in turn improving process‐related and proximal outcomes. Benefits to the HCP may be reduction in the required in‐visit time with the patient, potentially addressing provider time constraints. That said, our feedback panel reflected that PtDAs provided outside the context of a clinical visit may not be read or, if they are, may induce anxiety.

**Figure 2:**
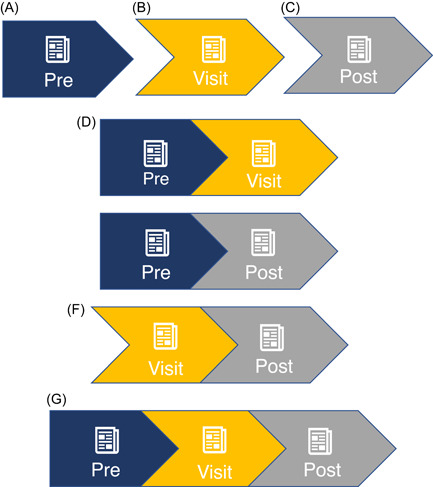
Permutations of single‐visit timing and frequency of PtDAs. Providers may choose to provide PtDAs one, two or three times during a single healthcare visit. (1) Diagrams (A–C) each represent one point of distribution. (2) Diagrams (D–F) each represent two points of distribution. (3) Diagram (G) represents three points of distribution. PtDA, patient decision aid.

**Figure 3:**
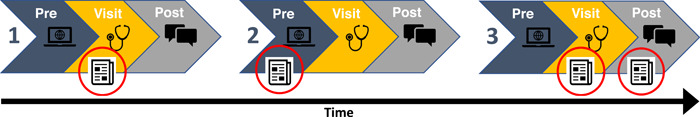
Example of cross‐visit frequency of PtDAs. This figure illustrates a series of three healthcare visits, each containing a ‘preparation’, ‘encounter’ and ‘processing’ stage. The PtDA icon, enclosed by a red circle, indicates the use of a PtDA at a given time point. In this example, PtDAs are delivered at the ‘encounter’ stage of Visit 1, the ‘preparation’ stage of Visit 2 and the ‘encounter’ and ‘processing’ stages of Visit 3. PtDA, patient decision aid.

We discuss the concepts of (1) timing, (2) single‐visit frequency and (3) cross‐visit frequency as they relate to improvement of L‐SDM. *Timing* refers to whether PtDAs are distributed in the preparation (previsit), encounter (in‐visit) or processing (postvisit) stages (see Figure 2A–C). Variation in timing can serve to emphasize a particular stage of the decision‐making process. For example, a previsit PtDA may allow the patient to discuss information with friends or family members and consolidate ideas before discussing with their HCP. Similarly, a postvisit PtDA allows the patient to continue deliberating at home, synthesizing the HCP's input with feedback from decision partners. Next, *single‐visit frequency* refers to the number of times a patient receives a PtDA surrounding any given healthcare visit. Patients may receive PtDAs at one, two or three of the delineated time points. Figure 2 shows all possible permutations of single‐visit PtDA dissemination, with diagrams D–F showing dissemination at two time points and diagram G reflecting the distribution of a PtDA at all three time points for a given visit. Finally, *cross‐visit frequency* assesses PtDA distributions across a series of visits. Figure 3 shows three distinct healthcare visits, each of which includes the three stages from Clayman et al.'s^7^ model. The figure shows an example sequence of cross‐visit PtDA distribution: (1) during the first visit, the provider uses a PtDA to initiate dialogue, (2) the patient receives a PtDA before the second visit to prime them to revisit the discussion and (3) the provider uses another in‐visit PtDA to resolve any unaddressed questions and provides a postvisit PtDA so that the patient can finalize his or her choice.

### 6.1 Exemplar: End‐of‐life care

We examine the application of PtDAs to L‐SDM for end‐of‐life decisions to demonstrate how understanding the integration of these two concepts can impact healthcare delivery. After providing background information on advance care planning (ACP), we discuss the current state of SDM in ACP and explore the potential for PtDAs in L‐SDM to improve outcomes.

End‐of‐life care can involve difficult decisions between aggressive life‐sustaining treatments or less invasive care with a primary focus on quality of life.^37^ The presence of multiple chronic conditions is associated with higher intensity, utilization and cost of end‐of‐life care.^41^, ^42^ Despite complex decisions, patients report not being adequately informed about potential consequences of life‐sustaining treatments.^43^ The process of ACP informs patients to better evaluate options for future care.

While 65%–87% of patients express a desire to participate in end‐of‐life treatment decisions, providers used SDM in fewer than half of palliative care visits.^44^ Focus groups of HCPs identified potential explanations for minimal use of SDM: some felt that providing information about these sensitive topics might signal giving up or defeat, or that making decisions against life‐sustaining treatment were contrary to their goal of saving lives.^45^ In addition, providers expressed concern that PtDAs—explicit patient‐facing tools to support SDM—were ‘devaluing’ their role in facilitating difficult conversations with patients. However, patients reported a preference for SDM early in their illness trajectory, noting in particular that they would like preparation for code status discussions.^45^

While decision partners may be present for any medical decision, their inclusion is especially relevant in the context of ACP. They may be participants and discussants, or they may serve as proxies for underage or incapacitated patients.^46^ As a result, these decision partners may have in‐depth conversations with the patient outside of the clinic or even be present for the medical visit itself. Additionally, patients and providers recommend an upstream approach to ACP, starting early in life or course of illness and evolving over time.^45^ Even though the patient may be residing in the hospital, processing and dialogue are likely to occur in the intervals between discussions with the provider. Taken together, L‐SDM is especially pertinent to end‐of‐life care.

For patients with advanced illness, PtDAs have been shown to increase their sense of empowerment and control by affirming their choices, encouraging future proactivity and strengthening motivation.^47^ Decision tools used in this context are a mix of previsit and in‐visit PtDAs.^40^ A focus group eliciting provider preferences found disparate views on optimal timing.^45^ For end‐of‐life care, it may be valuable to assess desired outcomes for the same PtDA distributed before, during or after the visit. Patients and caregivers also express interest in being more informed rather than less informed when it comes to ACP decisions.^44^ As such, a single PtDA may be insufficient to fully support L‐SDM, and identification of which permutation(s) of single‐visit frequency (Figure 2D–G) is most effective could enhance delivery of end‐of‐life care. With ACP, it is explicitly recommended that decisions be made across a series of medical encounters,^45^ with subsequent opportunity to revisit decisions. Incorporating PtDAs with cross‐visit frequency may decrease provider hesitation around broaching the topic by placing less pressure on a single conversation. As with other SDM contexts, it is important to integrate SDM for end‐of‐life care into workflows to decrease the burden on individual providers and clinics.^46^ Further research is needed to determine the optimal timing of dissemination for both patient and provider uptake and satisfaction.

## 7 CONCLUSION

PtDAs are an important element of SDM, but to date, there has been limited evidence for optimal frequency and timing of their use in L‐SDM. We propose a model for both testing and implementing L‐SDM that (a) highlights three timepoints of PtDA distribution, and (b) shows how these timepoints may be leveraged across visits to improve L‐SDM.

This model has multiple implications for future research. The design of PtDAs distributed outside of the visit may need to differ in content from in‐visit PtDAs to encourage engagement and reduce harm (i.e., anxiety related to presentation of information). Providers and healthcare systems may need to incentivize and reinforce review of PtDAs outside of the visit (e.g., encourage them to discuss with a decision partner, nurse or peer mentor), give patients options to choose the timing and frequency of receiving PtDAs and facilitate discussion of PtDAs external to clinical visits (e.g., via use of interactive online resources, secure messaging or telehealth).

Ultimately, the implications of evaluating optimal *timing* and *frequency* for PtDAs in L‐SDM are far‐reaching. By introducing, understanding and leveraging how these two factors impact the process of decision‐making—and the resulting choice—we may improve patient and provider outcomes.

## CONFLICT OF INTEREST

The authors declare no conflict of interest.

## Data Availability

Data sharing is not applicable to this article as no new data were created or analysed in this study.

## References

[1] Fraenkel L , McGraw S . What are the essential elements to enable patient participation in medical decision making? J Gen Intern Med. 2007;22(5):614‐619. 10.1007/s11606-007-0149-9 17443368 PMC1855272

[2] Barry MJ , Edgman‐Levitan S . Shared decision making—the pinnacle of patient‐centered care. N Engl J Med. 2012;366(9):780‐781. 10.1056/NEJMp1109283 22375967

[3] Charles C , Gafni A , Whelan T . Shared decision‐making in the medical encounter: what does it mean? (or it takes at least two to tango). Soc Sci Med. 1997;44(5):681‐692. 10.1016/S0277-9536(96)00221-3 9032835

[4] Fisher KA , Tan ASL , Matlock DD , Saver B , Mazor KM , Pieterse AH . Keeping the patient in the center: common challenges in the practice of shared decision making. Patient Educ Couns. 2018;101(12):2195‐2201. 10.1016/j.pec.2018.08.007 30144968 PMC6376968

[5] Bomhof‐Roordink H , Fischer MJ , van Duijn‐Bakker N , et al. Shared decision making in oncology: a model based on patients', health care professionals', and researchers' views. Psychooncology. 2019;28(1):139‐146. 10.1002/pon.4923 30346076

[6] Montori VM , Gafni A , Charles C . A shared treatment decision‐making approach between patients with chronic conditions and their clinicians: the case of diabetes. Health Expect. 2006;9(1):25‐36. 10.1111/j.1369-7625.2006.00359.x 16436159 PMC5060323

[7] Clayman ML , Gulbrandsen P , Morris MA . A patient in the clinic; a person in the world. Why shared decision making needs to center on the person rather than the medical encounter. Patient Educ Couns. 2017;100(3):600‐604. 10.1016/j.pec.2016.10.016 27780646

[8] Couët N , Desroches S , Robitaille H , et al. Assessments of the extent to which health‐care providers involve patients in decision making: a systematic review of studies using the OPTION instrument. Health Expect. 2015;18(4):542‐561. 10.1111/hex.12054 23451939 PMC5060794

[9] Williams D , Edwards A , Wood F , et al. Ability of observer and self‐report measures to capture shared decision‐making in clinical practice in the UK: a mixed‐methods study. BMJ Open. 2019;9(8):1‐10. 10.1136/bmjopen-2019-029485 PMC6701565 31427333

[10] Elwyn G , Frosch D , Thomson R , et al. Shared decision making: a model for clinical practice. J Gen Intern Med. 2012;27(10):1361‐1367. 10.1007/s11606-012-2077-6 22618581 PMC3445676

[11] Keij SM , van Duijn‐Bakker N , Stiggelbout AM , Pieterse AH . What makes a patient ready for shared decision making? A qualitative study. Patient Educ Couns. 2020;104(3):571‐577. 10.1016/j.pec.2020.08.031 32962880

[12] Hess EP , Coylewright M , Frosch DL , Shah ND . Implementation of shared decision making in cardiovascular care: past, present, and future. Circulation. 2014;7(5):797‐803. 10.1161/CIRCOUTCOMES.113.000351 25052074

[13] Elwyn G , Scholl I , Tietbohl C , et al. “Many miles to go …”: a systematic review of the implementation of patient decision support interventions into routine clinical practice. BMC Med Inform Decis Mak. 2013;13(S2):S14. 10.1186/1472-6947-13-S2-S14 24625083 PMC4044318

[14] Scalia P , Durand M‐A , Berkowitz JL , et al. The impact and utility of encounter patient decision aids: systematic review, meta‐analysis and narrative synthesis. Patient Educ Couns. 2019;102(5):817‐841. 10.1016/j.pec.2018.12.020 30612829

[15] Martínez‐Alonso M , Carles‐Lavila M , Pérez‐Lacasta MJ , Pons‐Rodríguez A , Garcia M , Rué M . Assessment of the effects of decision aids about breast cancer screening: a systematic review and meta‐analysis. BMJ Open. 2017;7(10):e016894. 10.1136/bmjopen-2017-016894 PMC5640065 28988175

[16] Søndergaard SR , Madsen PH , Hilberg O , Jensen KM , Olling K , Steffensen KD . A prospective cohort study of shared decision making in lung cancer diagnostics: impact of using a patient decision aid. Patient Educ Couns. 2019;102(11):1961‐1968. 10.1016/j.pec.2019.05.018 31129012

[17] Baethge C , Goldbeck‐Wood S , Mertens S . SANRA—a Scale for the Quality Assessment of Narrative Review articles. Res Integr Peer Rev. 2019;4:1. 10.1186/s41073-019-0064-8 30962953 PMC6434870

[18] Merker VL , Hyde JK , Herbst A , et al. Evaluating the impacts of patient engagement on health services research teams: lessons from the veteran consulting network. J Gen Intern Med. 2022;37(1):33‐41. 10.1007/s11606-021-06987-z 35349028 PMC8993982

[19] Rapley T . Distributed decision making: the anatomy of decisions‐in‐action: doctor‐patient relationships and distributed decision making. Sociol Health Illn. 2008;30(3):429‐444. 10.1111/j.1467-9566.2007.01064.x 18194358

[20] Bussey LG , Sillence E . The role of internet resources in health decision‐making: a qualitative study. Digit Health. 2019;5:205520761988807. 10.1177/2055207619888073 PMC6843735 31741741

[21] Epstein RM , Street RL . Shared mind: communication, decision making, and autonomy in serious illness. Ann Fam Med. 2011;9(5):454‐461. 10.1370/afm.1301 21911765 PMC3185482

[22] Joseph‐Williams N , Williams D , Wood F , et al. A descriptive model of shared decision making derived from routine implementation in clinical practice (‘Implement‐SDM’): qualitative study. Patient Educ Couns. 2019;102(10):1774‐1785. 10.1016/j.pec.2019.07.016 31351787

[23] Lown BA , Hanson JL , Clark WD . Mutual influence in shared decision making: a collaborative study of patients and physicians. Health Expect. 2009;12:160‐174. 10.1111/j.1369-7625.2008.00525.x 19236633 PMC5060484

[24] Kjos AL , Worley MM , Schommer JC . Medication information seeking behavior in a social context: the role of lay and professional social network contacts. Innov Pharm. 2011;2(4):2. 10.24926/iip.v2i4.246

[25] Rupert DJ , Gard Read J , Amoozegar JB , et al. Peer‐generated health information: the role of online communities in patient and caregiver health decisions. J Health Commun. 2016;21(11):1187‐1197. 10.1080/10810730.2016.1237592 27805496 PMC7315436

[26] Elwyn G , Frosch D , Volandes AE , Edwards A , Montori VM . Investing in deliberation: a definition and classification of decision support interventions for people facing difficult health decisions. Med Decis Making. 2010;30(6):701‐711. 10.1177/0272989X10386231 21088131

[27] Brackett C , Kearing S , Cochran N , Tosteson ANA , Blair Brooks W . Strategies for distributing cancer screening decision aids in primary care. Patient Educ Couns. 2010;78(2):166‐168. 10.1016/j.pec.2009.06.013 19665338

[28] Hsu C , Liss DT , Frosch DL , Westbrook EO , Arterburn D . Exploring provider reactions to decision aid distribution and shared decision making: lessons from two specialties. Med Decis Making. 2017;37(1):113‐126. 10.1177/0272989X16671933 27899745

[29] Hsu C , Liss DT , Westbrook EO , Arterburn D . Incorporating patient decision aids into standard clinical practice in an integrated delivery system. Med Decis Making. 2013;33(1):85‐97. 10.1177/0272989X12468615 23300204

[30] Dobler CC , Sanchez M , Gionfriddo MR , et al. Impact of decision aids used during clinical encounters on clinician outcomes and consultation length: a systematic review. BMJ Qual Saf. 2019;28(6):499‐510. 10.1136/bmjqs-2018-008022 PMC6561726 30301874

[31] Graham MM , James MT , Spertus JA . Decision support tools: realizing the potential to improve quality of care. Can J Cardiol. 2018;34(7):821‐826. 10.1016/j.cjca.2018.02.029 29861205

[32] Riikonen JM , Guyatt GH , Kilpeläinen TP , et al. Decision aids for prostate cancer screening choice: a systematic review and meta‐analysis. JAMA Intern Med. 2019;179(8):1072‐1082. 10.1001/jamainternmed.2019.0763 31233091 PMC6593633

[33] van Weert JCM , van Munster BC , Sanders R , Spijker R , Hooft L , Jansen J . Decision aids to help older people make health decisions: a systematic review and meta‐analysis. BMC Med Inform Decis Mak. 2016;16(1):45. 10.1186/s12911-016-0281-8 27098100 PMC4839148

[34] Violette PD , Agoritsas T , Alexander P , et al. Decision aids for localized prostate cancer treatment choice: systematic review and meta‐analysis: prostate cancer treatment choice. CA Cancer J Clin. 2015;65(3):239‐251. 10.3322/caac.21272 25772796

[35] Coronado‐Vázquez V , Canet‐Fajas C , Delgado‐Marroquín MT , Magallón‐Botaya R , Romero‐Martín M , Gómez‐Salgado J . Interventions to facilitate shared decision‐making using decision aids with patients in primary health care: a systematic review. Medicine. 2020;99(32):e21389. 10.1097/MD.0000000000021389 32769870 PMC7593011

[36] Stacey D , Légaré F , Lewis K , et al. Decision aids for people facing health treatment or screening decisions. Cochrane Database Syst Rev. 2017;4:001431. 10.1002/14651858.CD001431.pub5 PMC6478132 28402085

[37] Baik D , Cho H , Masterson Creber RM . Examining interventions designed to support shared decision making and subsequent patient outcomes in palliative care: a systematic review of the literature. Am J Hosp Palliat Med. 2019;36(1):76‐88. 10.1177/1049909118783688 PMC6056336 29925244

[38] Tapp D , Blais M‐C . Evaluation of decision support tools for patients with advanced cancer: a systematic review of literature. Palliat Support Care. 2019;17(3):356‐364. 10.1017/S1478951518000512 30168410

[39] Jones LA , Weymiller AJ , Shah N , et al. Should clinicians deliver decision aids? further exploration of the statin choice randomized trial results. Med Decis Making. 2009;29:468‐474. 10.1177/0272989X09333120 19605885

[40] Scholl I , LaRussa A , Hahlweg P , Kobrin S , Elwyn G . Organizational‐ and system‐level characteristics that influence implementation of shared decision‐making and strategies to address them—a scoping review. Implement Sci. 2018;13(1):40. 10.1186/s13012-018-0731-z 29523167 PMC5845212

[41] McDermott CL , Engelberg RA , Sibley J , Sorror ML , Curtis JR . The association between chronic conditions, end‐of‐life health care use, and documentation of advance care planning among patients with cancer. J Palliat Med. 2020;23(10):1335‐1341. 10.1089/jpm.2019.0530 32181689 PMC7523017

[42] Wagner E , Patrick DL , Khandelwal N , et al. The influence of multimorbidity on health care utilization at the end of life for patients with chronic conditions. J Palliat Med. 2019;22(10):1260‐1265. 10.1089/jpm.2018.0349 30964382

[43] Becker C , Lecheler L , Hochstrasser S , et al. Association of communication interventions to discuss code status with patient decisions for do‐not‐resuscitate orders: a systematic review and meta‐analysis. JAMA Netw Open. 2019;2(6):e195033. 10.1001/jamanetworkopen.2019.5033 31173119 PMC6563579

[44] Bélanger E , Rodríguez C , Groleau D . Shared decision‐making in palliative care: a systematic mixed studies review using narrative synthesis. Palliat Med. 2011;25(3):242‐261. 10.1177/0269216310389348 21273220

[45] Jones J , Nowels C , Kutner JS , Matlock DD . Shared decision making and the use of a patient decision aid in advanced serious illness: provider and patient perspectives. Health Expect. 2015;18(6):3236‐3247. 10.1111/hex.12313 25439268 PMC5810681

[46] Lund S , Richardson A , May C . Barriers to advance care planning at the end of life: an explanatory systematic review of implementation studies. PLoS One. 2015;10(2):e0116629. 10.1371/journal.pone.0116629 25679395 PMC4334528

[47] Matlock DD , Keech TAE , McKenzie MB , Bronsert MR , Nowels CT , Kutner JS . Feasibility and acceptability of a decision aid designed for people facing advanced or terminal illness: a pilot randomized trial: a decision aid for people with advanced illness. Health Expect. 2014;17(1):49‐59. 10.1111/j.1369-7625.2011.00732.x 22032553 PMC5060696

